# Individual and social determinants of early sexual activity: A study of gender-based differences using the 2018 Canadian Health Behaviour in School-aged Children Study (HBSC)

**DOI:** 10.1371/journal.pone.0238515

**Published:** 2020-09-03

**Authors:** Naomi Gazendam, Kathryn Cleverley, Nathan King, William Pickett, Susan P. Phillips

**Affiliations:** 1 Department of Family Medicine, Queen's University, Kingston, Ontario, Canada; 2 Public Health Sciences, Queen's University, Kingston, Ontario, Canada; University of Ghana, GHANA

## Abstract

**Introduction:**

Early sexual activity, often defined as initiation before the age of 16, is a risk behaviour associated with negative health outcomes in adulthood. The objective of this study was to explore links between early sexual activity and individual and contextual characteristics in Canadian youth, and whether these differ for girls and boys.

**Methods:**

Data were from the 2018 Canadian Health Behaviour in School-aged Children (HBSC) survey administered in classrooms across Canada to students in grades 6 to 10 (ages 11 to 16). The sample includes the 7882 students in grades 9 and 10 who were asked about sexual activity. Individual and contextual measures used included emotional well-being, socioeconomic status, participation in team sports, body image, social media use, family structure, and family support. Descriptive data overall and separately for girls and boys are presented, followed by Poisson regression models to estimate relative risks and associated 95% confidence intervals for strength of associations between characteristics and early sexual activity. Models were adjusted for clustering by school using generalised estimating equations.

**Results:**

Overall, contextual factors i.e. disrupted family structure or low family support were the characteristics most strongly associated with early sexual activity. Among boys there was an incremental and strong relationship between hours spent in organised sport and early sexual activity. Among girls, poorer body image, lower socioeconomic status, and higher social media use aligned most strongly with early sexual activity.

**Conclusion:**

Persistent gender stereotypes appear to underlie differences in individual and contextual factors associated with adolescents’ sexual behaviour. Findings from this exploratory analysis may be of benefit to subsequent researchers, policy makers and those who care for youth.

## Introduction

The World Health Organization (WHO) identifies positive sexual health and healthy views of one’s sexuality as imperative for physical and mental well-being [1]. In Europe and North America, sexual initiation often occurs during adolescence [2] and can be a marker and a predictor of risks such as coercion or abuse, inconsistent use of contraception, increased incidence of sexually transmitted infections (STI’s), unplanned pregnancy, substance abuse, subsequent sexual violence, or increased numbers of sexual partners [3–8].

Timing of sexual debut is heavily influenced by personal and social circumstances and, particularly, parenting and education. At the individual level, the well-studied relationship between poor mental health and early sexual activity is strong, particularly among adolescent girls [2, 9–14]. This relationship is hypothesised to be bidirectional; girls struggling with mental health are more likely to engage in early sexual activity and early sexual activity appears to increase depressive symptoms. Depressed youth may have impaired social relationships, be less motivated to protect themselves, have diminished self-efficacy which negatively impacts their ability to resist pressure, and may attempt to alleviate emotional isolation and distress via sexual intimacy, further complicating poor mental health [4, 10, 15]. Sexual activity amongst youth may be hastened by peer pressure [14, 16] heightened emotional arousal, sensation seeking, and reward orientation [4]. The stage termination hypothesis, suggesting that youth can experience disrupted functioning when they initiate a developmental stage prior to mastering necessary skills, may also be applicable [9]. Early sexual activity can contribute to guilt or shame, and external cultural messaging, which influence how youth interpret their sexual experience and, in turn, diminish mental health further [12]. Sexual relationships may ultimately change existing affiliations between friends and family, adding to stress [12].

Multiple interconnected social factors have been identified as explanatory. Higher family income or socioeconomic status (SES) appears to protect against early sexual initiation [17]. Regardless of household income, living with both parents, more intense parental monitoring, and stronger communication appear to delay actual and intended sexual activity [1, 2, 17–22]. Conversely, youth living in what are referred to as disrupted families (e.g. single parent) tend to initiate sexual activity earlier. Less parental supervision or observation of different patterns of sexual behaviour than those experienced in intact families may contribute to this [17]. Findings about the relationship between physical activity and early sexual debut are contradictory, with one study documenting higher levels of sexual activity for students participating in sports [23] and another suggesting female high performance athletes are less likely than non-athletes to report having sexual intercourse [24]. In contrast, athletic participation for boys may strengthen gender scripts and sexual opportunism often reflected in the language of sexual conquest used among male athletes [25]. The 'jock' identity and culture, particularly prevalent among male athletes, is associated with sexual risk behaviours [25]. Finally, findings about screen time and early sexual activity are limited. A Dutch study found that more computer and television use/watching was associated with earlier sexual initiation [26]. There is, however, minimal research focusing on the specific impact of social media use.

It may be that predictors and meanings of early sexual activity differ for girls and boys. Girls generally reach emotional maturity earlier than males. Although this might imply that earlier sexual behaviour is more developmentally appropriate amongst females, a cultural double standard rewards boys for this sexual activity while shaming girls [10, 27]. The impact of self-esteem on early sexual activity is also gendered. A longitudinal American study identified early sexual activity to be more likely among girls with low self-esteem and boys with high self-esteem [28]. Positive body image is an important component of well-being and self-esteem, linked to early sexual activity among boys but non-activity in girls [27]. A stronger association between being sexually active and being depressed was reported amongst girls than boys [13] who also did not experience increased depressive symptoms after sexual initiation [2].

Although both individual and contextual factors, relationships between these, and gender differences are all likely entwined as predictors and outcomes of early sexual activity, actual evidence is limited. Whether aspects of mental health are predictors and/or outcomes of early sexual activity is not known. The link between early sexual activity and emotional wellness, a marker of mental health in adolescents, also remains unexplored. Emotional well-being is a subjective indicator of life satisfaction and happiness [29], of positive feelings about life. Such life satisfaction appears to promote strong mental health, the ability to create meaningful relationships, to engage in fulfilling activities, and to feel optimistic. Emotional well-being indicates quality of life in youth, and shapes present and future experiences. Our aim, therefore, was to explore the connected nature of individual and contextual factors that lead to, but at times are shaped by early (and possibly risky) sexual activity, and whether these differ for girls and boys. Of particular interest were characteristics that are amenable to change via individual or more systemic interventions.

## Methods

### Conceptual model

**Fig 1:**
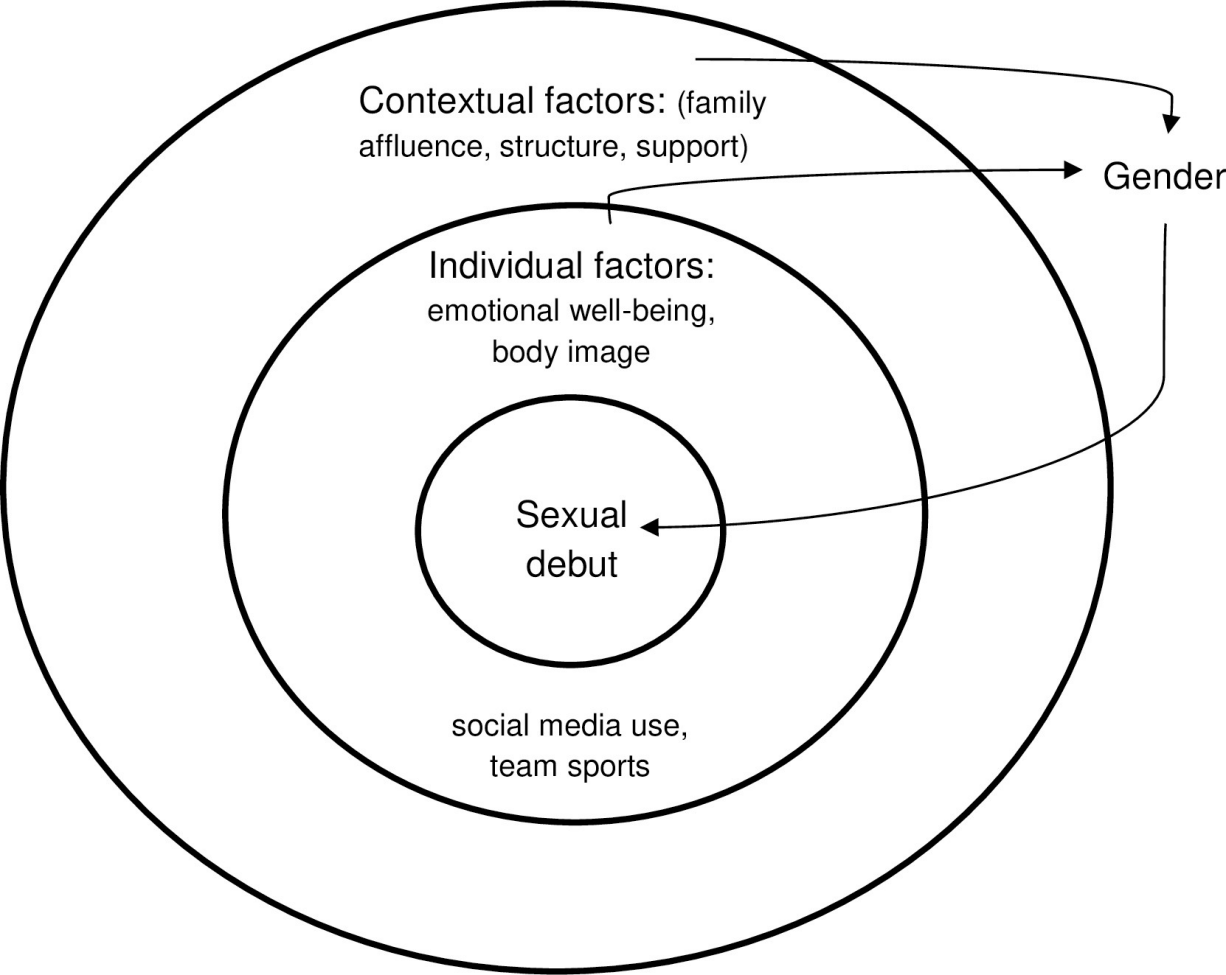
Conceptual framework pre-analyses: Exploratory model of factors underlying early sexual activity.

We hypothesised that there were numerous possible individual and contextual precursors of early sexual activity (Fig 1). For individual factors we considered that their effect was likely bi-directional, whereas contextual characteristics were more likely predictors of early sexual debut than results of it. We further hypothesised that the impact of each characteristic could be modified by sex/gender, that is, by whether the participant was male or female and by social norms and expectations aligned with each of these two categories. We are aware of the risks of categorising by sex with its inherent assumption of homogeneity within categories and heterogeneity across these. For all these reasons and to identify intersecting aspects of individual and social circumstances with sex, we have disaggregated results for girls and boys.

### Study population

Data for this study come from the 2018 Canadian Health Behaviour in School-aged Children (HBSC). A self-report questionnaire was administered in classrooms to collect information on individual and social circumstances, health, and health-related behaviours. The HBSC surveys students in grades 6 to 10 (typically ages 11 to 16) from all Canadian provinces and territories. Excluded from the study are the <7% of Canadian children from private schools, home school situations, schools on First Nation or Inuit reserves, youth not in school and those who are incarcerated. This study only used responses from grade 9 and 10 students as only they were asked about sexual activity.

After removing schools that opted out of the sexual activity questions the available grade 9–10 sample numbered 7,882. Restricting to youth with complete responses to all variables included in the multivariable analysis left us with a total sample of 6,154 (Male = 2,848; Female = 3,199; Neither = 107); weighted n = 5,456 (Male = 2,505; Female = 2,858; Neither = 92).

### Ethics

Ethics approval was granted by the Public Health Agency of Canada (REB-2013-0022), and Queen’s University Research Ethics Board (6027003). Participation in the study was voluntary and consent based on local protocol was obtained from administrators, parents and students. All data were anonymized prior to our accessing them.

### Study variables

Early Sexual Activity was defined as having sexual intercourse at the age of 15 years or younger, based on the standard definition of early sexual activity as that occurring before the age of 16 [2, 9, 30]. Specific questions asked were: (1)“*Have you ever had sexual intercourse (sometimes this is called “making love”*, *“having sex”*, *or “going all the way”)*?*”*, and (2) “*How old were you when you had sexual intercourse for the first time*”. Youth who reported never having had sexual intercourse or that their first sexual experience was at the age of 16 years or older were included in the ‘No’ category. Early sexual activity as defined in this paper refers only to sexual intercourse, not other types of sexual activity. HBSC data do not further describe early sexual activity as, for example consensual, nor is information about partner(s) available.

Age in years was used. Youth self-identified as either “*Male*”, “*Female*”, or “*Neither term describes me*”.

Emotional well-being was measured using the Cantril Ladder. Respondents position themselves on a drawing of a ladder when asked to rate their life satisfaction from 0 “Worst possible life” to 10 “Best possible life”. Categories for analysis were ‘Highest’ (10), ‘High’ (8–9), ‘Moderate’ (6–7), or ‘Low’ (0–5). Amongst 11–15 year-olds the Cantril ladder has been repeatedly validated and used as a single robust measure of emotional well-being [31]. For body image, youth reported how much they agree or disagree with the following statement: “*I would change how I look if I could*.” Five response options were re-categorised into three groups: Disagree, Neither agree nor disagree, or Agree.

Social media use was measured with the item “*How many hours a day*, *in your free time*, *do you usually spend… Using electronic devices such as computers*, *tablets (like iPad) or smartphones for other purposes (e*.*g*., *homework*, *emailing*, *tweeting*, *Facebook*, *chatting*, *surfing the internet)*?”. The response options (“*None at all*”, “*About half an hour a day*”, “*About 1 hour a day*”, “*About 2 hours a day*”, “*About 3 or more hours a day*”) were dichotomised into <3 hours versus ≥3 hours a day.

For organised sport participation youth reported how many hours a week (none at all, about ½ hour, about 1 hour, about 2 hours, about 3 hours, about 4 or more hours) they usually spend doing “*Organised sports that are not part of gym class (e*.*g*., *school sports team*, *hockey team*, *dance practice*, *swim club)*". Total hours per week was calculated and grouped into: <1 hour, About 1 to 3 hours, or 4 hours or more.

The following were the contextual indicators utilised. Relative family affluence was measured with the item “*How well off do you think your family is*?” Five response options were collapsed into three categories: ‘Well off’, ‘Average’, and ‘Not well off’. Family support was measured using a 4-item scale [32] (α = 0.91): “*My family really tries to help me*”, “*I get the emotional help and support I need from my family*”, “*I can talk about my problems with my family*”, “*My family is willing to help me make decisions*” (response options: 1 = Very strongly disagree to 7 = Very strongly agree). A composite score was modelled using approximate tertiles based on the full HBSC sample distribution. Family Structure was measured by asking youth to identify who they live with all or most of the time, and categorised as: “Intact” (live with both natural parents) or “Disrupted” family (live with one natural parent and no step-parent, one natural parent and a step-parent, or neither natural parent) [30].

### Statistical analysis

SAS version 9.4 was used for all analyses. The sample was first described overall, and then by sex using frequencies and means (standard deviations). The Rao-Scot chi-square test, adjusting for clustering by school, was used to test for differences in the distribution of categorical variables between boys and girls. A mixed effects linear regression model that adjusted for clustering by school was used to test for differences in mean age between boys and girls. For descriptive analyses all values were weighted, so that they represent the prevalence estimates in Canadian adolescents.

Poisson regression models were used to estimate bivariate and adjusted relative risks and associated 95% confidence intervals, examining the associations between selected covariates and early sexual intercourse. Models were adjusted for clustering by school using generalised estimating equations (GEE).

For the stratified analyses by sex, youth who identified as “neither term describes me” were excluded because of small numbers (n = 92), and lack of statistical power.

## Results

Socio-demographic characteristics of the 5456 grade 9 and 10 students included in the Canadian 2018 HBSC survey can be seen in Table 1, reported collectively and separately for boys and girls. Relative to girls, boys were more likely to have intact families from whom they received support, were less likely to use social media for more than 3 hours a day, and reported overall higher life satisfaction. Girls reported lower SES and poorer body image than did boys. Although overall the excess of girls who had had sex may be of importance it was not statistically significant (19.3% versus 17.8%, p = 0.35). Among those who were sexually active at the earliest ages (</ = 12 or 13), the proportion of boys exceeded that of girls (10.9% versus 3.5%, p < .01 and 15.7% versus 12.4%, p = .34).

**Table 1:** Description of sample overall and by sex, 2018 HBSC study (n = 6,154, weighted n = 5,456).

			Full Sample (n = 5,456)		Male (n = 2,505)		Female (n = 2,858)		Neither (n = 92)	
			n	(%)	n	(%)	n	(%)	n	(%)
**Age, years, Mean(SD)**			15.3	(0.7)	15.4	(0.7)	15.3	(0.7)	15.4	(1.0)
**Relative family affluence**^**a**^										
	Well off		2913	(53.4)	1444	(57.6)	1430	(50.0)	39	(42.7)
	Average		2120	(38.9)	891	(35.6)	1194	(41.8)	35	(35.1)
	Not well off		422	(7.7)	170	(6.8)	234	(8.2)	18	(19.2)
**Family structure**^**a**^										
	Intact family		4010	(73.5)	1927	(76.9)	2027	(70.9)	55	(59.6)
	Disrupted family		1446	(26.5)	578	(23.1)	831	(29.1)	37	(40.4)
**Family support**^**a**^										
	High		1630	(29.9)	856	(34.2)	768	(26.9)	7	(7.1)
	Moderate		1810	(33.2)	865	(34.5)	918	(32.1)	28	(30.2)
	Low		2015	(36.9)	785	(31.3)	1172	(41.0)	58	(62.7)
**Life satisfaction**^**a**^										
	Highest (10)		328	(6.0)	214	(8.6)	111	(3.9)	3	(3.1)
	High (8–9)		2098	(38.5)	1170	(46.7)	915	(32.0)	12	(13.2)
	Moderate (6–7)		1913	(35.1)	784	(31.3)	1103	(38.6)	27	(28.8)
	Low (0–5)		1117	(20.5)	337	(13.5)	730	(25.5)	50	(54.8)
**Would change how I look If I could**^**a**^										
	Disagree		2077	(38.1)	1218	(48.6)	838	(29.3)	22	(23.4)
	Neither agree nor disagree		1066	(19.5)	523	(20.9)	519	(18.2)	23	(24.9)
	Agree		2313	(42.4)	764	(30.5)	1501	(52.5)	48	(51.7)
**Organised sport participation**										
	Less than 1 hr		2341	(42.9)	1023	(40.8)	1267	(44.3)	51	(55.3)
	About 1 to 3 hrs		1613	(29.6)	764	(30.5)	821	(28.7)	29	(31.2)
	4 or more hrs		1502	(27.5)	719	(28.7)	770	(27.0)	12	(13.5)
**Social media use, hrs/day**^**a**^										
	Less than 3 hrs		3776	(69.2)	1832	(73.1)	1889	(66.1)	54	(59.1)
	3 or more hrs		1680	(30.8)	673	(26.9)	969	(33.9)	38	(40.9)
**Early sexual intercourse, Yes**			1028	(18.8)	446	(17.8)	551	(19.3)	32	(34.3)
	Age at first intercourse^a^									
		≤12	76	(7.4)	49	(10.9)	19	(3.5)	8	(26.3)
		13	146	(14.2)	70	(15.7)	68	(12.4)	8	(24.1)
		14	333	(32.4)	108	(24.2)	220	(40.0)	5	(16.6)
		15	473	(46.0)	219	(49.2)	243	(44.1)	10	(23.0)

All values are weighted.

^a^Rao-Scot Chi-Square test comparing the distribution in male and female students p < .05.

In bivariate analyses, each of age, body image, family support, family structure, physical activity, social media use, and perception of family affluence were significantly correlated with early sexual activity for boys, girls, or both. Of the individual level factors lowest life satisfaction and least positive body image were significant among girls, only, team sports were of incremental significance among boys, and greater social media use was significant for both although more so for girls. Of contextual factors considered the strongest associations were as follows: disrupted family structure (both girls and boys); lowest level of family support; and decrements in affluence (both, girls > boys).

Age, sex and all significant factors from bivariate analyses were included in the multivariable, sex-stratified Poisson regression (S1 Table). Older age aligned with greater likelihood of sexual initiation for all. The impact of all other factors considered, whether individual or contextual varied with sex. Greater social media use showed the strongest of all measured associations with early sexual activity for girls (RR = 1.43, 95% CI: 1.25–1.64) and a lesser alignment for boys (RR = 1.18, 95% CI: 1.00–1.40). Girls with poor body image were more likely to have engaged in early sexual activity (RR = 1.22; 95% CI: 1.01–1.47) whereas among boys, body image bore no relationship with early sex. Conversely, for boys the relationship between time spent in team sports and early sexual activity was incremental and the strongest predictor identified (RR = 2.13, 95% CI: 1.73–2.63) while of no significance for girls. The measure of emotional well-being was life satisfaction. For boys, only findings for those reporting high (but not the highest) levels were significant (RR = 0.76, CI: 0.59–0.98). Among girls there was a stepwise increase in early sexual activity with decreasing life satisfaction that did not reach statistical significance.

Of the contextual characteristics examined, disrupted family structure had a strong effect overall, and, particularly for boys (RR = 1.53, CI: 1.31–1.78) among whom only team sport participation was stronger. This measure was, nevertheless, one of the most significant for girls as well (RR = 1.41, CI: 1.23–1.62), second only to social media use. As with family disruption, support from families was important for both boys (RR = 1.45; 95% CI: 1.22–1.72) and girls (RR = 1.35; 95% CI: 1.12–1.63). Lowest family affluence, however, was predictive of early sexual activity only among girls (RR = 1.25, 95% CI: 1.01–1.55).

## Discussion

We have explored individual and contextual factors that predict and may be influenced by early sexual activity. Their impact appears to be modified by sex/gender in ways that generally align with theory about gender roles. Overall, the strongest associations identified for both boys and girls were contextual, between disrupted family structure or low family support and early sexual activity. However, when girls and boys were examined separately, among boys, the strongest effect arose from participation in organised sport whereas for girls, lower family affluence, higher social media use, and poorer body image were key. The connection between life satisfaction and sexual initiation was not of importance, but, of note, among girls the relationship was inverse (highest life satisfaction was associated with lowest levels of sexual activity) while for boys it was J-shaped (intermediate life satisfaction aligned with lowest rates).

Much of what we found was congruent with existing evidence, but there were surprising results, particularly aspects of both contextual and individual characteristics that have not been previously examined and that hint at persistent and pervasive gender stereotypes and traditional roles. In keeping with others' findings, both disrupted family structure and poor family support were associated with early sexual activity for all and, particularly for boys [1, 2, 3, 17–20]. Perhaps adolescents from disrupted families experience less adult supervision, an explanation put forward by others [17], but also one that stereotypes single parents. Sex/gender differences observed were in keeping with others' findings that parental monitoring is a protective factor for all and, particularly, for boys [22]. Perhaps girls experience more parental control despite family structure which, in turn, could ameliorate the impact of family disruption on them. Again, gender stereotypes reinforced in parenting could underlie this finding. Youth without close family ties may seek these in sexual relationships [17]. Girls' greater ability to develop emotional ties with friends regardless of family closeness may be a stereotype but may also preclude their seeking out connection via sexual activity [33]. Conversely, boys might substitute sexual for emotional connections they stereotypically are less likely to form.

Among girls but not boys, and in keeping with other's research, there was a strong relationship between lower affluence and early sexual activity [17, 18]. The subjective measure of affluence was closely and directly correlated with answers to whether participants had their own bedroom, adding validity to the measure. It is possible that lower SES families may have fewer resources to provide adult supervision for adolescents or to fund extracurricular activities that occupy their children's time [17]. Perhaps the greater impact of perceived SES among girls arises from intersections among gender, SES and self-confidence.

As hypothesised, girls who spent more time on social media were more likely to have engaged in early sexual activity, a relationship that was less pronounced in boys. Social media use brings with it increased exposure to sexualised messages and images [34] that may break down barriers to engaging in sexual activity. High social media use might be a marker of loneliness or a need for peer approval among girls. This same need may also predispose to girls' early sexual activity in an attempt to fit in, gain popularity or achieve emotional fulfillment and positive peer recognition [16].

It could be that a negative impact of social media on girls’ body image, self-esteem and early sex are all interconnected. Adolescent girls may be particularly sensitive to images on social media that glorify specific body types. Images depicting thin and stereotypically sexually appealing body shapes, combined with peer pressure, perpetuate girls', more than boys' feelings of dissatisfaction with their own bodies [35] and may adversely impact girls’ confidence and self-esteem. In keeping with this, body image of female participants was poor relative to males, and girls with poorer body image were more likely to have engaged in early sexual activity. This relationship is likely bidirectional. Adolescent girls with poor self-esteem may search for acceptance and validation in the form of sexual activity [28], lack self-confidence and be less able to refuse sexual advances, viewing them as a confirmation of acceptance. At the same time, such girls may also monitor their bodies and be self-conscious during sexual activity, possibly augmenting feelings of inadequacy [36]. The interconnection of lower self-confidence, poor self esteem and body image, and earlier sexual activity among girls but not boys likely arises from and suggests the perpetuation of traditional gender roles [37].

Girls with lower life satisfaction trended toward engaging in early sexual activity. Life satisfaction is an indicator of emotional well-being, and likely also has a bidirectional relationship with early sexual activity. Again, girls may seek to engage in early sexual activity thinking it will provide validation, then feel worse about themselves and less emotionally well afterwards. Conversely, boys with the highest life satisfaction were more likely to have engaged in early sexual activity than were those reporting the two middle levels of this measure, perhaps seeking sexual activity for pleasure rather than emotional need. Stereotypically, engaging in sexual activity elevates boys' status whereas girls tend to be shamed for such activity [10]. Boys reporting intermediate levels of life satisfaction are less likely to engage in early sexual activity, a finding not seen among girls. Perhaps girls anticipate that sexual activity will bring validation and belonging. This might also explain why boys who have not engaged in early sexual activity appear to be more content than girls.

Existing research on the impact of sports on teens' behaviours and risk taking is contradictory [23, 24]. We found no relationship between team sport participation and early sex for girls but a strong and incremental alignment among boys. Among boys, participation may solidify historical gender narratives that support masculinised notions of sexuality. 'Locker room talk' reflects a milieu where using one's position to exploit women is encouraged. Athletic participation can foster culturally constructed gender scripts among boys [25]. Jock identity encourages sexual opportunism and boys may use their peer status as athletes to coerce [25]. The social and cultural capital that enables boys to take sexual risks, seems to be entwined in traditional masculinity [25].

Several limitations are inherent in using cross-sectional data. We are unable to determine causality or directions of associations. Students may have been sensitive to answers considered socially desirable. Emotional well-being was measured at the time of study rather than before or at the time of first sexual intercourse. Although the questionnaire was meant to be completed anonymously, individually, and privately, the classroom setting may have influenced responses, particularly when school personnel were in the room. Participants were asked if they had ever had sexual intercourse, which was explained as "making love," "having sex," or "going all the way”. This question does not determine whether sexual intercourse was consensual or coercive, the nature of the relationship, age difference with partner(s), or associated substance use. Students may not have understood terminology used or how to categorise anything but penetrative sex, and may have assumed only heterosexual activity was to be reported. When students were asked how many hours a day they used electronic devices, the questions included use for homework and mentioned only some networking platforms, making it difficult to separate out 'social' media use from, for example, online gaming or schoolwork. There were no questions in the HBSC that addressed hook-up culture via apps such as Tinder. In order to assess family cohesion, students were asked how many meals a week their families have together. The question included breakfast, a meal that may be a weaker indicator of cohesion than is supper.

**Fig 2:**
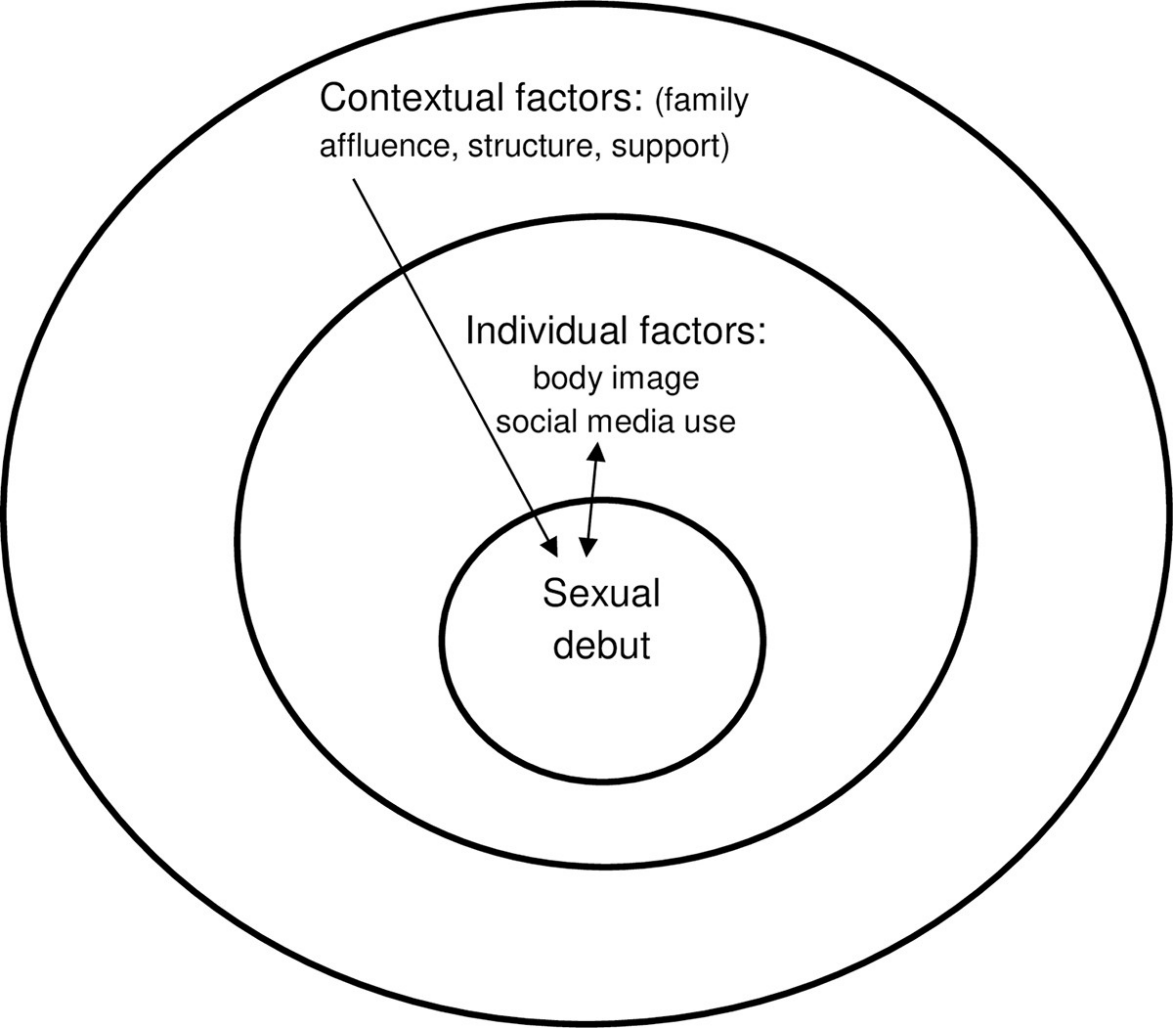
Conceptual framework post-analysis: Exploratory model of factors underlying early sexual activity in girls.

**Fig 3:**
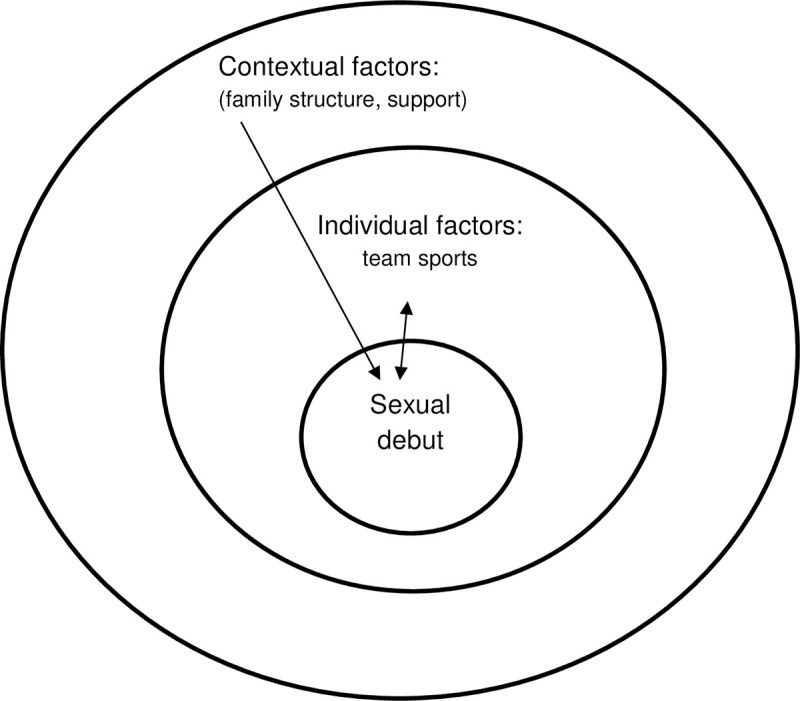
Conceptual framework post-analysis: Exploratory model of factors underlying early sexual activity in boys.

Based on findings we refined our initial conceptual model to better describe the moderating effect of gender, and to hypothesise about the bidirectional effect of the individual level factors considered (Figs 2 and 3). In summary, individual characteristics such as high social media use, poor body image, and team sport participation are associated with early sexual activity but this association is modified by gender. Among boys, early sexual activity may be driven by societal definitions and expectations of masculinity given correlations with sports and positive life satisfaction. For girls, early sex may offer the allure of gaining social acceptance and fitting in although might not deliver on this and, as a result may lead to poorer self-image and well-being. Contextual indicators of family structure and support align with less sexual activity for all youth. In contrast, the observed interplay between gender and affluence, could operate at both the contextual and individual levels. Among girls, perceived low affluence may increase vulnerability and a need for validation and acceptance.

Overall, our findings suggest the persistent influence of gender stereotypes on adolescents’ sexual behaviour and on the emotional fall-out for girls from that behaviour. Changing what many consider to be a risky behaviour, that is, early sexual debut, will require addressing these underlying gender roles. It is tempting to recommend policies and education programs, and consider when these might be better delivered to each group alone (i.e. boys separate from girls) versus collectively. Unfortunately, this approach is simplistic and not evidence-based [37]. Despite decades of research and writing about the harms of traditional gender roles, they seem to persist. Perhaps examining what has enabled role shifts in some countries might provide guidance about next steps, as might explicit and systemic top-down indications of change in perceptions, policies and practices that enshrine gender equity.

## Supporting information

S1 Table Bivariate and adjusted associations between covariates and sexual activity, by sex. (PDF)
