# Supplementary material for: Individual and social determinants of early sexual activity: A study of gender-based differences using the 2018 Canadian Health Behaviour in School-aged Children Study (HBSC)
Source: PLoS One. 2020 Sep 3;15(9):e0238515. doi: 10.1371/journal.pone.0238515 (PMC7470420; doi:10.1371/journal.pone.0238515)
Supplement: S1 Table — (PDF) [file pone.0238515.s001.pdf]

S1 Table. Bivariate and adjusted associations between covariates and sexual activity, by sex

	**Male** (n= 2,887)							**Female** (n= 3,236)						
				Bivariate		Adjusted[a]					Bivariate		Adjusted[a]	
	n total	n Yes	(%Yes)	RR	(95% CI)	RR	(95% CI)	n total	n Yes	(%Yes)	RR	(95% CI)	RR	(95% CI)
**Age, years**	/	/	/	1.42	(1.26-1.60)	1.41	(1.27-1.57)	/	/	/	1.49	(1.32-1.69)	1.45	(1.29-1.63)
**Organized Sport Participation**														
Less than 1 hr	1148	190	(16.6)	1.00	ref	1.00	ref	1345	300	(22.3)	1.00	ref	1.00	ref
About 1 to 3 hrs	809	164	(20.3)	1.29	(1.06-1.58)	1.48	(1.21-1.83)	889	199	(22.4)	0.99	(0.83-1.18)	1.17	(0.99-1.39)
4 or more hrs	891	247	(27.7)	1.80	(1.47-1.21)	2.13	(1.73-2.63)	965	178	(18.5)	0.88	(0.76-1.03)	1.14	(0.98-1.32)
**Social Media Use, hrs/day**														
Less than 3 hrs	2069	401	(19.4)	1.00	ref	1.00	ref	2064	353	(17.1)	1.00	ref	1.00	ref
3 or more hrs	779	200	(25.7)	1.24	(1.03-1.49)	1.18	(1.00-1.40)	1135	324	(28.6)	1.59	(1.38-1.83)	1.43	(1.25-1.64)
**Relative family affluence**														
Well off	1567	310	(19.8)	1.00	ref	1.00	ref	1572	281	(17.9)	1.00	ref	1.00	ref
Average	1073	231	(21.5)	1.08	(0.91-1.26)	0.97	(0.82-1.14)	1355	306	(22.6)	1.21	(1.05-1.41)	0.99	(0.85-1.15)
Not well off	208	60	(28.9)	1.50	(1.17-1.91)	1.22	(0.96-1.55)	272	90	(33.1)	1.84	(1.48-2.27)	1.25	(1.01-1.55)
**Family Structure**														
Intact	2035	369	(18.1)	1.00	ref	1.00	ref	2155	374	(17.4)	1.00	ref	1.00	ref
Disrupted	813	232	(28.5)	1.62	(1.38-1.89)	1.53	(1.31-1.78)	1044	303	(29.0)	1.67	(1.44-1.90)	1.41	(1.23-1.62)
**Family Support**														
High (Q1) (3)	889	152	(17.1)	1.00	ref	1.00	ref	823	130	(15.8)	1.00	ref	1.00	ref
Moderate	999	198	(19.8)	1.13	(0.94-1.34)	1.14	(0.96-1.36)	1012	172	(17.0)	1.12	(0.91-1.38)	0.98	(0.80-1.20)
Low (Q3) (1)	960	251	(26.2)	1.58	(1.34-1.87)	1.45	(1.22-1.72)	1364	375	(27.5)	1.81	(1.51-2.18)	1.35	(1.12-1.63)
**Would change how I look**														
Disagree (3)	1331	279	(21.0)	1.00	ref	1.00	ref	822	125	(15.2)	1.00	ref	1.00	ref
Neither agree nor disagree	588	111	(18.9)	0.97	(0.80-1.17)	0.91	(0.74-1.11)	607	108	(17.8)	1.16	(0.96-1.39)	1.12	(0.92-1.35)

Agree	929	211	(22.7)	1.12	(0.95-1.32)	0.99	(0.83-1.18)	1770	444	(25.1)	1.59	(1.33-1.90)	1.22	(1.01-1.47)
**Life satisfaction**														
Highest (10)	277	69	(24.9)	1.00	ref	1.00	ref	143	20	(14.0)	1.00	ref	1.00	ref
High (8-9)	1228	222	(18.1)	0.71	(0.56-0.91)	0.76	(0.59-0.98)	1025	157	(15.3)	1.08	(0.71-1.66)	1.03	(0.66-1.59)
Moderate (6-7)	922	189	(20.5)	0.80	(0.63-1.02)	0.82	(0.63-1.07)	1144	227	(19.8)	1.37	(0.87-2.15)	1.12	(0.69-1.81)
Low (0-5)	421	121	(28.7)	1.19	(0.92-1.54)	1.10	(0.83-1.46)	887	273	(30.8)	2.17	(1.42-3.33)	1.44	(0.90-2.30)

Note: ‘Yes’ refers to having engaged in sexual intercourse before the age of 16

[a]adjusted for other covariates in the table, Unweighted Poisson regression
